# Author Correction: The solute carrier SLC9C1 is a Na^+^/H^+^-exchanger gated by an S4-type voltage-sensor and cyclic-nucleotide binding

**DOI:** 10.1038/s41467-020-18023-5

**Published:** 2020-08-19

**Authors:** F. Windler, W. Bönigk, H. G. Körschen, E. Grahn, T. Strünker, R. Seifert, U. B. Kaupp

**Affiliations:** 1 Center of Advanced European Studies and Research (caesar), Department Molecular Sensory Systems, Ludwig-Erhard-Allee 2, 53175 Bonn, Germany; 2 Marine Biological Laboratory, 7 MBL Street, Woods Hole, 02543 MA USA; 3 University Hospital Münster, Center of Reproductive Medicine and Andrology, Albert-Schweitzer-Campus 1, Geb. D11, 48149 Münster, Germany; 4 University of Bonn, Life & Medical Sciences Institute (LIMES), Carl-Troll-Str. 31, 53115 Bonn, Germany

**Keywords:** Ion channels, Membrane biophysics, Molecular biophysics, Ion channels

Correction to: *Nature Communications* 10.1038/s41467-018-05253-x, published online 18 July 2018.

In the original version of this Article, some traces in Fig. 5c were improperly scaled which resulted in a wrong representation of the data. In particular, the traces for 1 pM Speract, the red trace for 100 pM Speract and the red trace for 1 nM Speract were improperly scaled. This has now been corrected in the PDF and HTML versions of the Article.

