# Supplementary material for: The solute carrier SLC9C1 is a Na+/H+-exchanger gated by an S4-type voltage-sensor and cyclic-nucleotide binding
Source: Nat Commun. 2018 Jul 18;9:2809. doi: 10.1038/s41467-018-05253-x (PMC6052114; doi:10.1038/s41467-018-05253-x)
Supplement: Supplementary file 1 — Supplementary Information [file 41467_2018_5253_MOESM1_ESM.pdf]

Supplementary Information

The solute carrier SLC9C1 is a Na^+/H^+-exchanger gated by an S4-type voltage-sensor and cyclic-nucleotide binding

Windler et al.

Supplementary Figures 1-7

Supplementary Figure 8: Uncropped capture of immunoblot (Fig. 6a).

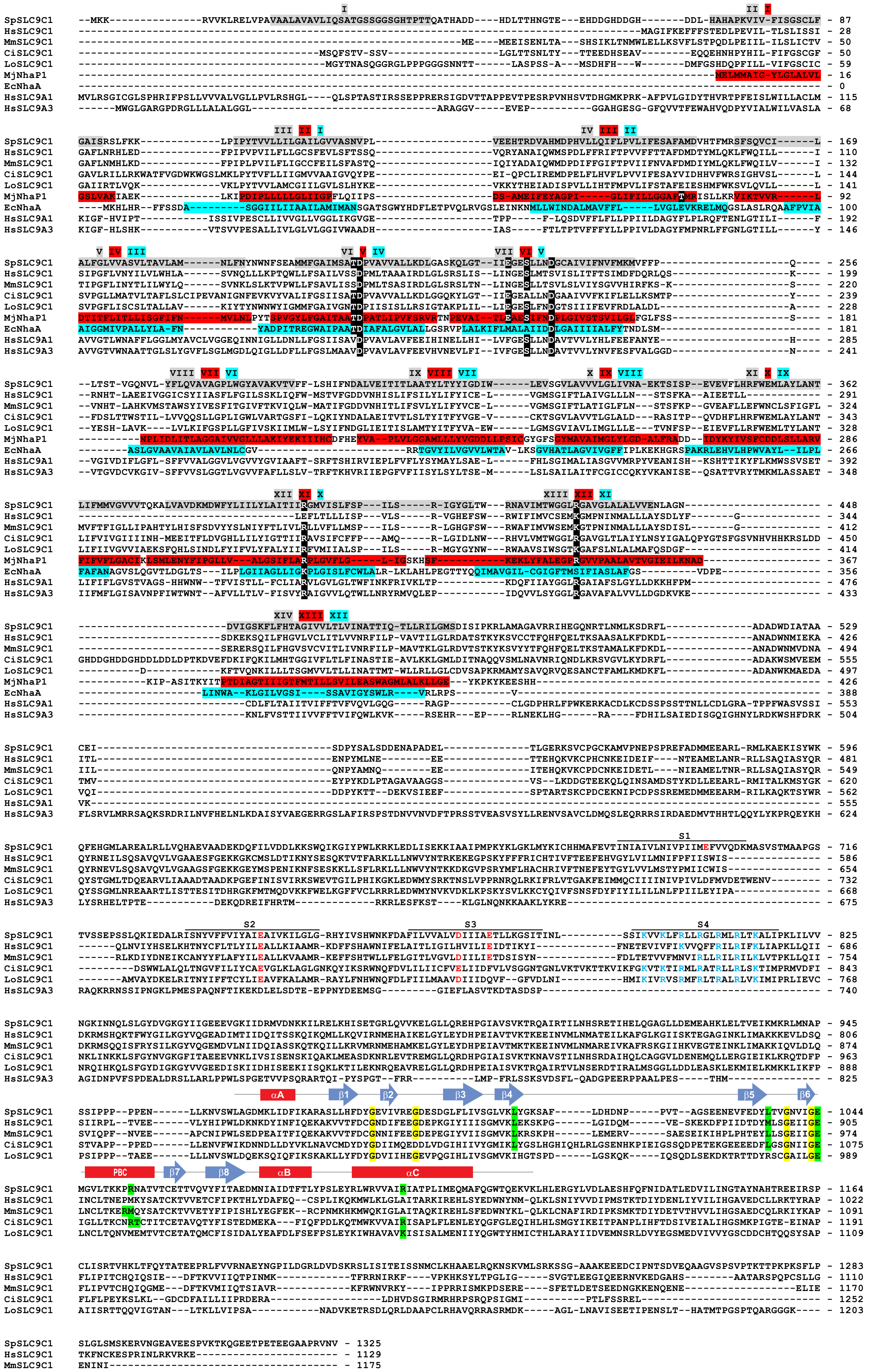

Supplementary Figure 1. Sequence Comparison of SLC9C1 from Different Species with Classical Na^+/H^+ Exchangers. Sequence comparison of SLC9C1 from multiple species with *Methanocaldococcus jannaschii* NhaP1 (*Mj*NhaP1), *Escherichia coli* NhaA (*Ec*NhaA), and human SLC9A1 (NHE1) and SLC9A3 (NHE3). SLC9C1 sequences include *S. purpuratus* (*Sp*SLC9C1), *H. sapiens* (*Hs*SLC9C1), *M. musculus* (*Mm*SLC9C1), ciona *C. intestinalis* (*Ci*SLC9C1), and spotted gar *L. oculatus* (*Lo*SLC9C1). Grey bars indicate transmembrane domains I-XIV of *Sp*SLC9C1 exchanger domain as predicted by hydropathy analysis. Red and blue bars indicate transmembrane domains I-XII from *Mj*NhaP1 and I-XII from *Ec*NhaA. Conserved amino-acid residues of the Na^+-binding site (shown in Fig. 1b) and amino-acid residues involved in exchange activity are marked. Black lines indicate S1-S4 of the voltage-sensor domain (VSD) as shown in Fig. 1c. The C-terminus of the SLC9C1 protein contains a cyclic nucleotide-binding domain (CNBD) (see Fig. 1d).

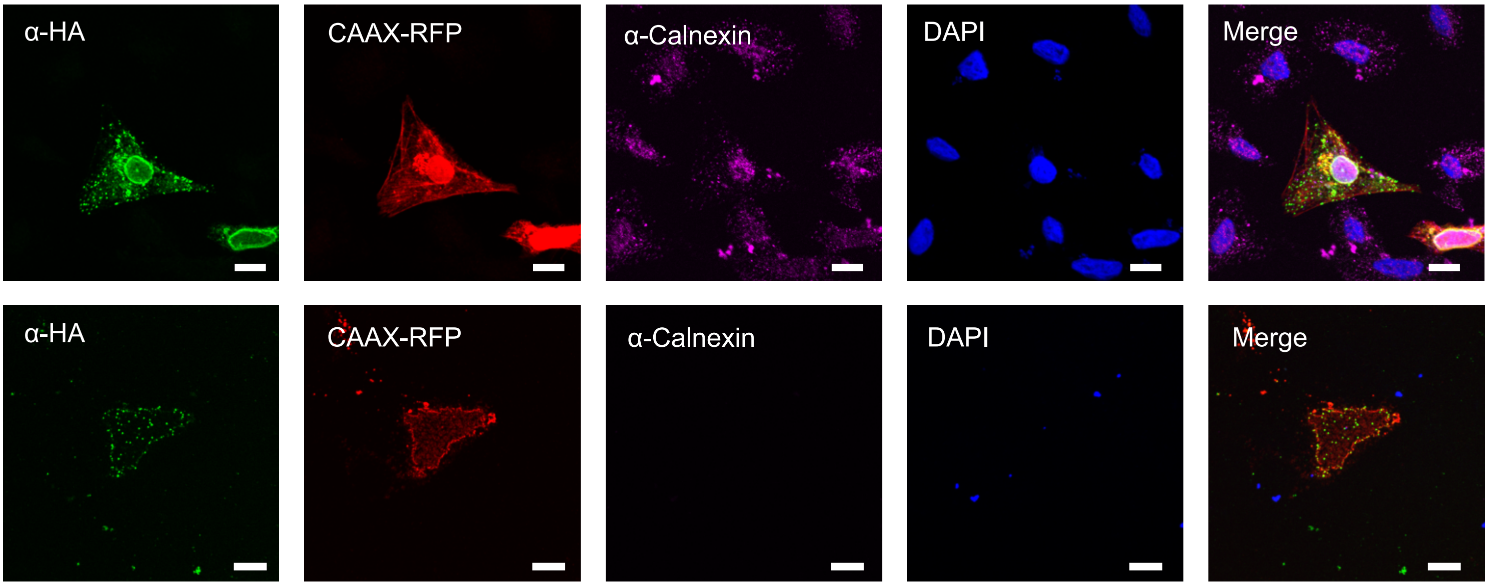

Supplementary Figure 2. Subcellular localization of *Sp*SLC9C1-HA in CHO cells. *Sp*SLC9C1-HA was probed with antibodies against the HA-tag (green), the ER was labelled with an antibody against Calnexin (magenta). As marker for plasma membrane, cells additionally expressed CAAX-RFP (red). The nucleus was stained with DAPI (blue). Upper panels: whole CHO cells. Lower panels: a short sonication pulse was applied to CHO cells such that only plasma membrane attached to the coverslip remained. Staining against the HA tag indicates plasma membrane localization of *Sp*SLC9C1-HA. Scale bars represent 10 µm.

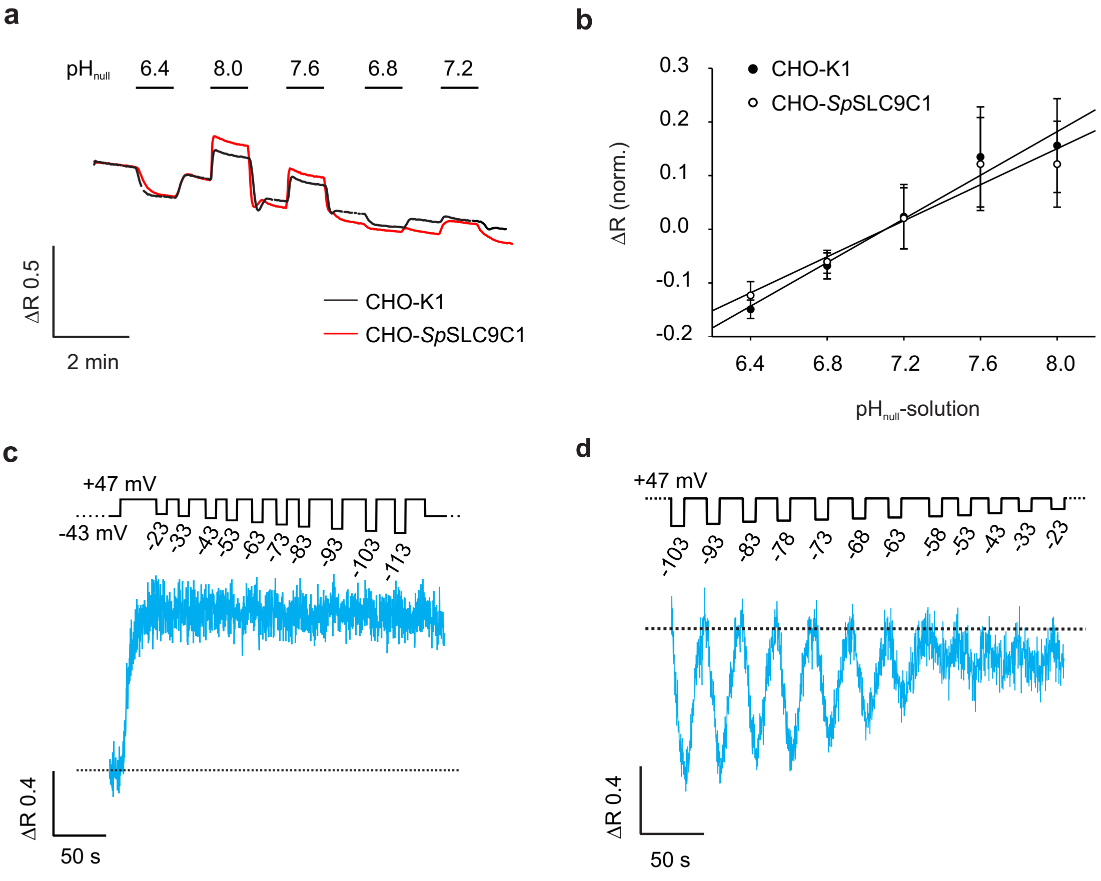

Supplementary Figure 3. Experimental controls for single-cell fluorimetry. (a) Representative measurements of pH_i changes imposed by pH_{null} solutions onto *Sp*SLC9C1- and non-transfected CHO cells. By comparison of baseline fluorescence with the subsequent change in pH_i, resting pH (pH_{rest}) can be estimated. If pH_{rest} is more acidic or basic than the pH imposed by the pH_{null} solution, perfusion results in an alkalization and acidification, respectively. The changes in pH_i imposed by pH_{null} solutions by and large are similar for control cells (black) and *Sp*SLC9C1 cells (red). **(b)** Quantification from 30 cells per pH_{null} value reveals no significant differences in buffer capacity and pH_{rest} (pH_i = 7.1) between *Sp*SLC9C1 and non-transfected CHO cells. **(c)** CHO cells expressing the human Hv1 proton channel without *Sp*SLC9C1. Initial depolarization of +47 mV alkalizes the cell until steady-state is reached. A voltage protocol from -23 to -113 mV shows no Na^+/H^+ exchange activity ($[Na^+]_i$ 14 mM; $[Na^+]_o$ 0 mM; pH_i = 7.2; pH_o = 7.4). **(d)** The voltage dependence of *Sp*SLC9C1 activation was probed by voltage steps ranging from -103 mV to -23 mV ($V_{1/2}$ = -68.4 mV). Hv1 was activated at +47 mV. All values are summarized in Table 1 ($[Na^+]_i$ 14 mM; $[Na^+]_o$ 0 mM; pH_i = 7.2; pH_o = 7.4).

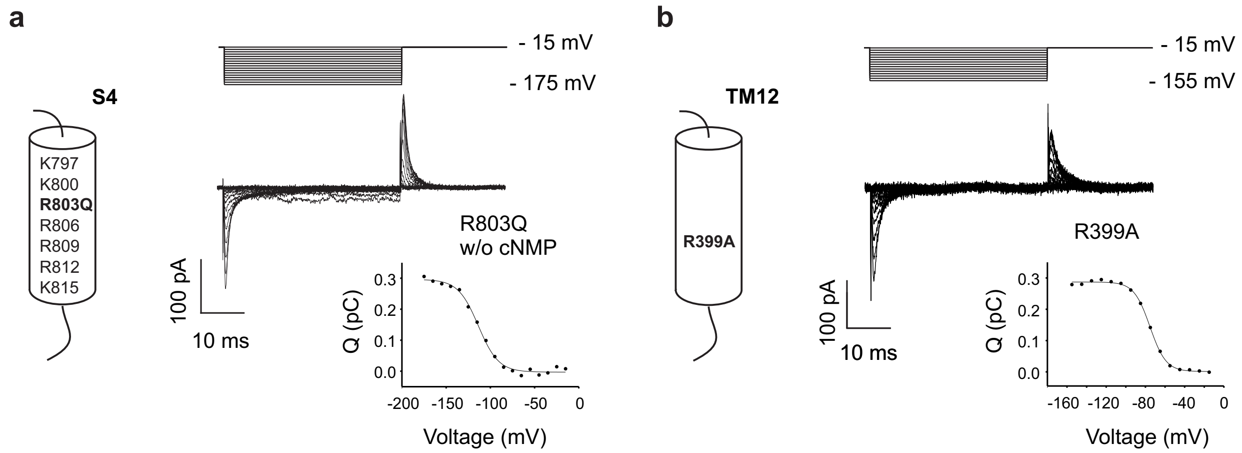

Supplementary Figure 4. Gating currents of *Sp*SLC9C1-R803Q and *Sp*SLC9C1-R399A mutants. (a) Scheme of the S4 segment in the voltage-sensing domain (VSD). The third positively charged amino acid was neutralized by a point mutation (R803Q). Representative gating currents and voltage dependence of the *Sp*SLC9C1-R803Q mutant. **(b)** Localization of the R399A mutation in TM12 of the *Sp*SLC9C1 exchanger domain. Representative gating currents and voltage dependence of the *Sp*SLC9C1-R399A mutant.

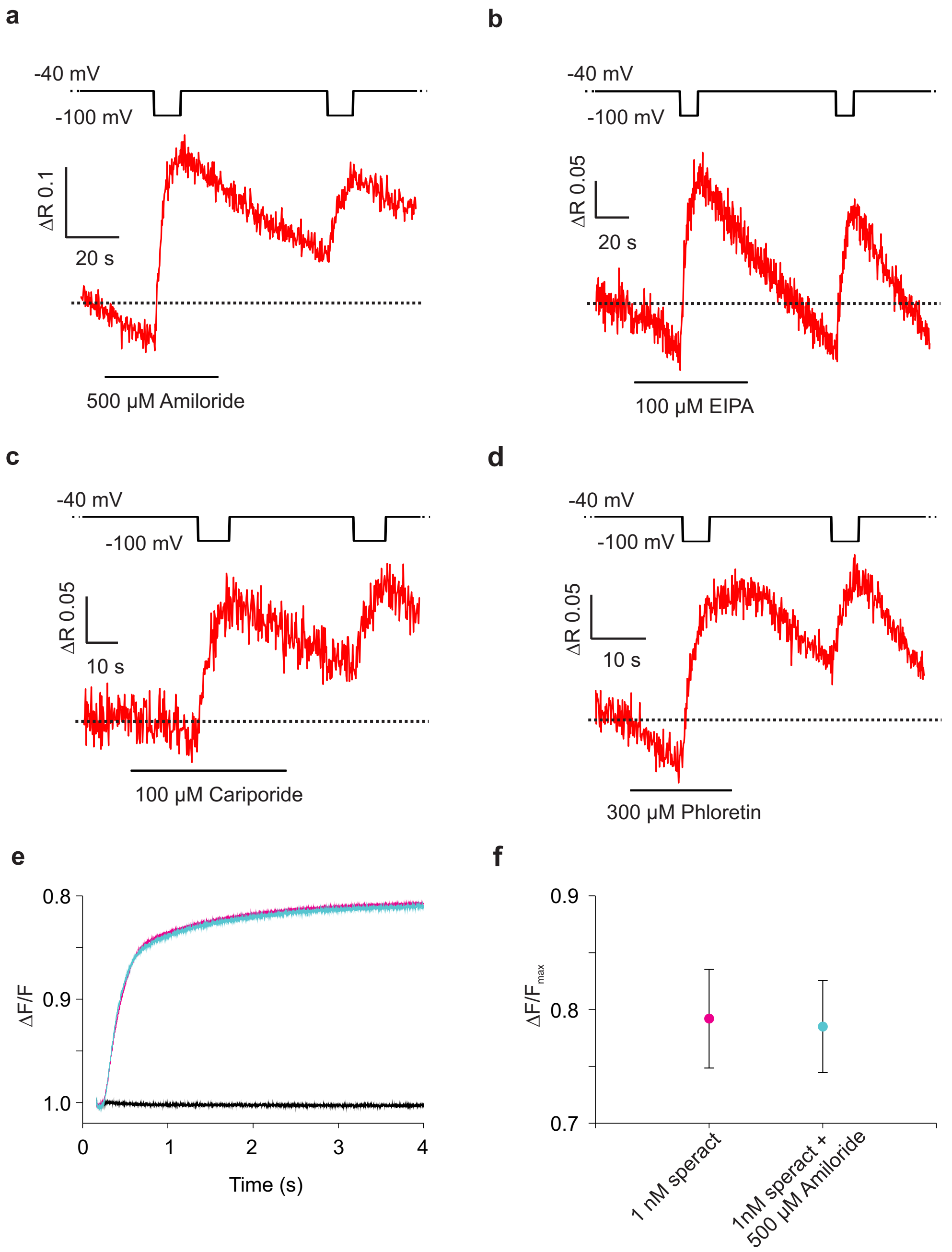

Supplementary Figure 5. Generic blockers do not inhibit *Sp*SLC9C1 exchange activity. *Sp*SLC9C1-CHO cells were stimulated with two 10-s voltage pulses to -100 mV (V_{hold} = -40 mV). During the first voltage pulse, cells were perfused with **(a)** 500 µM amiloride, **(b)** 100 µM EIPA, **(c)** 100 µM cariporide, and **(d)** 300 µM phloretin in ES. We observed exchange activity in presence of all four substances tested. Due to rundown of *Sp*SLC9C1 activity with time, the second voltage pulse elicited a slightly smaller alkalization. ($[Na^+]_o$ 140 mM; $[Na^+]_i$ 14 mM; pH_i = 7.2; pH_o = 7.4). **(e)** Alkalinization of sperm induced by 1 nM speract monitored by pHrodo Red in the absence (magenta trace) and presence (turquoise trace) of amiloride (500 µM). Black trace represents a control in the absence of speract stimulation. **(f)** Maximal change of fluorescence of pHrodo Red during stimulation with 1 nM speract in the absence (magenta) and presence (turquoise) of amiloride (500 µM) (n = 5).

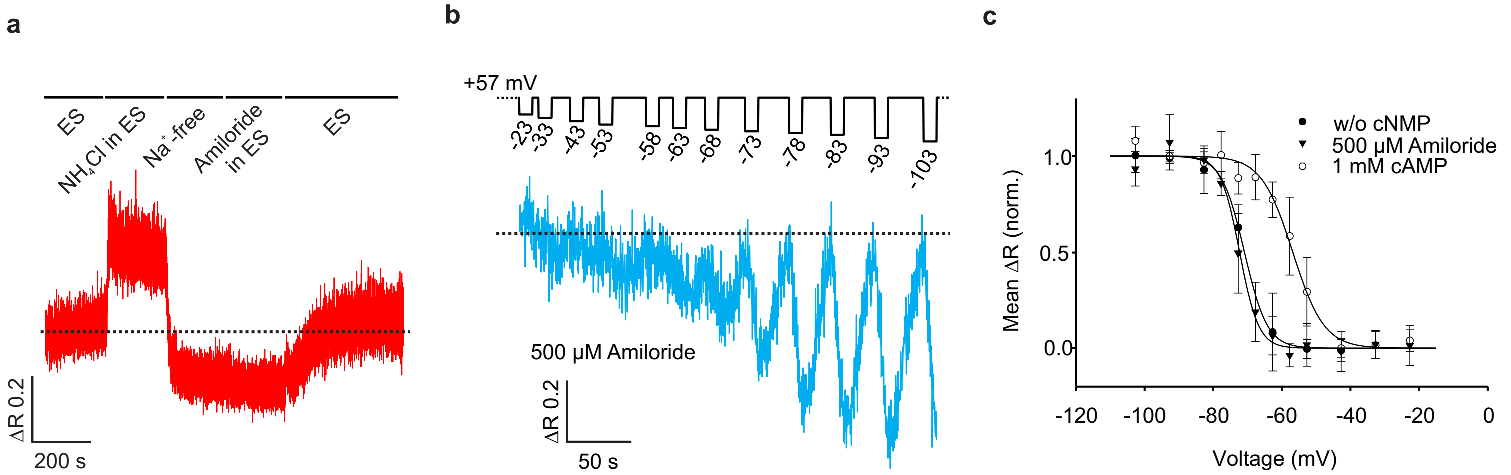

Supplementary Figure 6. Endogenous Na^+/H^+ exchange does not interfere with *Sp*SLC9C1 activity in CHO cells. (a) Acid-load assay in non-transfected CHO cells. A 200 s perfusion with 20 mM ammonium chloride (NH_4Cl) in ES alkalizes the cell line. During the subsequent perfusion with Na^+-free ES, the cell acidifies. Amiloride (500 µM) prevents the recovery from acidification. During the washout of amiloride, the pH_i returns to baseline values due to endogenous Na^+/H^+ exchange activity. **(b)** Voltage dependence of *Sp*SLC9C1 activation was probed by voltage steps ranging from -23 mV to -103 mV in the presence of amiloride (500 µM) ($V_{1/2}$= -72.7 mV) ($[Na^+]_i$ 14 mM; $[Na^+]_o$ 0 mM; pH_i = 7.2; pH_o = 7.4). **(c)** Normalized ΔR values were plotted against V_m to yield the $V_{1/2}$ values by a fit with the Boltzmann equation ($V_{1/2}$ = -73.3 ± 2.2 mV, *s* = 2.8 ± 1.1 mV, n = 3). Data were obtained without cAMP present. Amiloride (500 µM) does not affect the voltage dependence of *Sp*SLC9C1 activity. Mean values are summarized in Table 1.

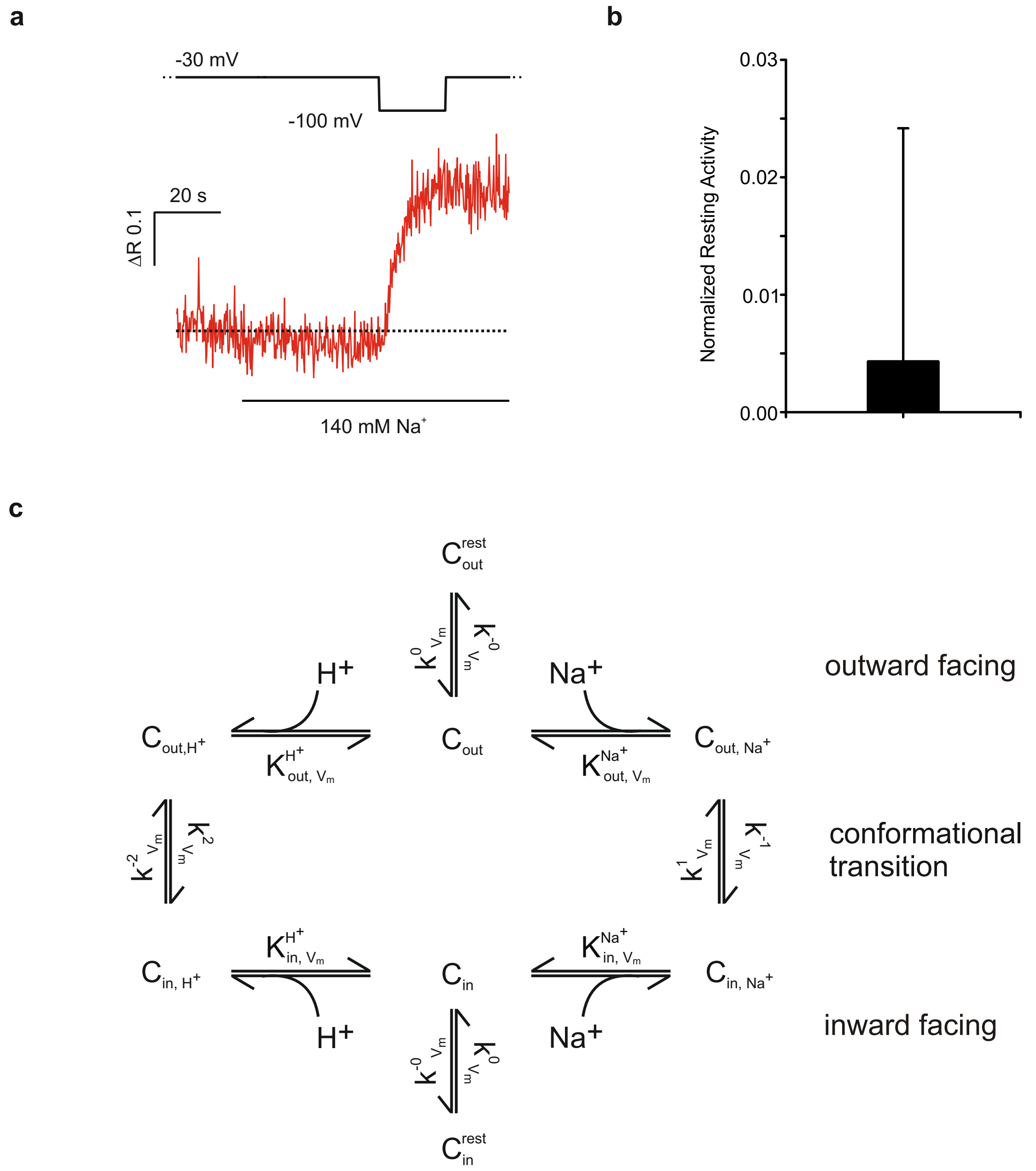

Supplementary Figure 7. Resting Activity of *Sp*SLC9C1. (a) At -30 mV and in the presence of 500 µM amiloride, cells were first perfused with symmetrical Na^+ solutions and in the absence of a pH gradient ($[Na^+]_i = [Na^+]_o = 14$ mM; $pH_i = pH_o = 7.2$). Exchange of the extracellular solution to standard ES ($[Na^+]_o$ 140 mM, $pH_o = 7.4$, see bar) was performed to elicit the resting activity of *Sp*SLC9C1 recorded by the BCECF dye. A subsequent voltage step to -100 mV maximally activated Na^+/H^+ exchange. **(b)** The slopes of the time course of fluorescence during perfusion with ES at -30 mV were background corrected by those at symmetrical conditions and normalized by those at -100 mV in ES. Mean value and standard deviation of 5 experiments. **(c)** Putative kinetic model of exchanger activity. Extended kinetic scheme of exchange activity: voltage either controls the rates of substrate access or binding or the rate of the conformational change.

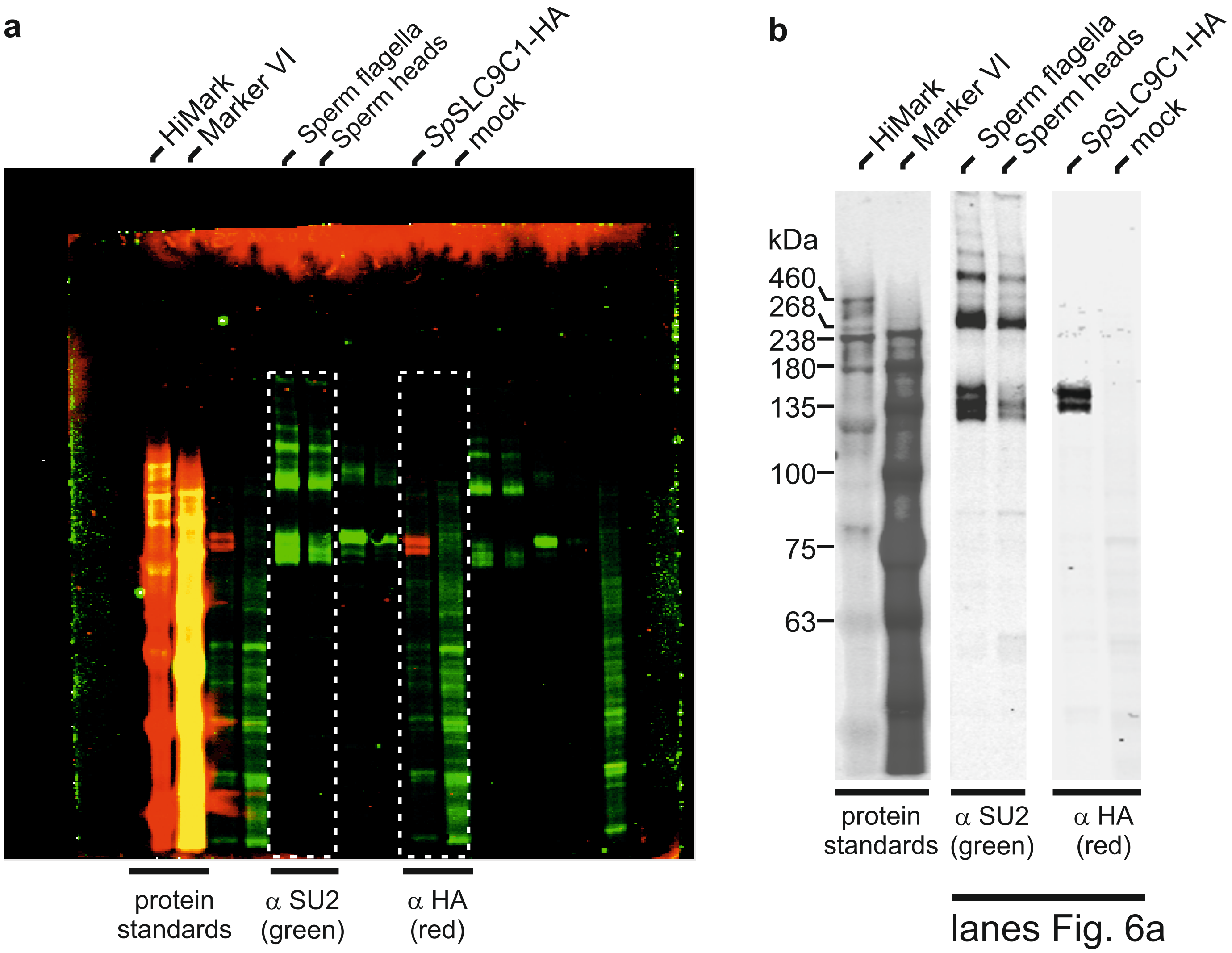

Supplementary Figure 8. Uncropped capture of immunoblot (Fig. 6a). (a) The colorized TIFF file was exported from the Application Software Version 3.0 of ODYSSEY infrared imaging system (LI-COR biosciences). Cropped areas are marked by white boxes. **(b)** Stainings of α SU2 antibody and α HA antibody were extracted from the green and the red channel of the colorized TIFF file, respectively, converted to grey scales and inverted using Photo-PaintX6 software (Corel Corporation). Contrast has been adjusted finally to achieve an appropriate final figure 6a. Sizes of protein bands were assigned using the fluorescence of the marker bands of the HiMark™ Pre-stained Protein Standard (Thermo Fisher Scientific, # LC5999) and the pre-stained Marker VI (Applichem, # A8889).
